# HIX003209 promotes vascular smooth muscle cell migration and proliferation through modulating miR-6089

**DOI:** 10.18632/aging.103079

**Published:** 2020-05-27

**Authors:** Xiaofeng Shi, Shuang Pan, Li Li, Yongqi Li, Wei Ma, Han Wang, Caiming Xu, Lei Li, Dong Wang

**Affiliations:** 1 Department of Emergency, Tianjin First Center Hospital, Tianjin 300192, People’s Republic of China; 2 Department of Physiology, School of Basic Medicine, Jinzhou Medicine University, Jinzhou 121000, Liaoning, People’s Republic of China; 3 Clinical Nutrition Department, The Second Affiliated Hospital of Dalian Medical University, Dalian 116027, Liaoning, People’s Republic of China; 4 Graduate School of Comprehensive Human Sciences, University of Tsukuba, Tsukuba 3050005, Japan; 5 Department of Anatomy, Dalian Medical University, Dalian 116044, Liaoning, People’s Republic of China; 6 Department of Vascular Surgery, Dalian University Affiliated Xinhua Hospital, Dalian 116021, Liaoning, People’s Republic of China; 7 Department of General Surgery, The First Affiliated Hospital, Dalian Medical University, Dalian 116011, Liaoning, People’s Republic of China; 8 Department of Vascular Surgery, The Second Affiliated Hospital of Dalian Medical University, Dalian 116027, Liaoning, People’s Republic of China; 9 Neurosurgery Department, The Second Affiliated Hospital of Dalian Medical University, Dalian 116027, Liaoning, People’s Republic of China

**Keywords:** atherosclerosis, lncRNAs, HIX003209, miR-6089

## Abstract

Accumulating references have showed that long noncoding RNAs (lncRNAs) act important roles in the development of human diseases. The role and expression of HIX003209 remains unclear in the pathogenesis of atherosclerosis. We showed that HIX003209 expression was upregulated in atherosclerotic coronary tissues compared to normal coronary artery samples. HIX003209 was overexpressed in vascular smooth muscle cells (VSMCs) induced by inflammatory mediators including tumor necrosis factor-α(TNF-α), ox-LDL and latelet-derived growth factor-BB (PDGF-BB). Ectopic expression of HIX003209 enhanced cell growth and migration and induced inflammatory mediators secretion such as interleukin 6 (IL-6), TNF-α and IL-1β in VSMCs. Furthermore, we showed that miR-6089 was downregulated in atherosclerotic coronary tissues compared to normal coronary artery samples. There was a negative association between expression of HIX003209 and miR-6089 in atherosclerotic coronary tissues. MiR-6089 expression was decreased in VSMCs induced by inflammatory mediators including TNF-α, ox-LDL and PDGF-BB. Dual luciferase analysis showed that miR-6089 overexpression decreased luciferase activity of HIX003209 WT-type 3’-UTR but not the mut-type 3’-UTR. Overexpression of HIX003209 suppressed the expression of miR-6089 in VSMCs. Ectopic expression of HIX003209 induced cell growth, migration and the secretion of inflammatory mediators via regulating miR-6089 expression. These data suggested that HIX003209 promoted VSMCs proliferation, migration and the secretion of inflammatory mediators partly via regulating miR-6089.

## INTRODUCTION

Atherosclerosis is one main cause of cerebral infarction, peripheral vascular disease and coronary heart disease [1–4]. Its etiology and pathogenesis are complex and not well clear. A lot of risk factors such as macrophages accumulation, pro-inflammatory cytokines production vascular smooth muscle (VSMCs) or endothelial cells dysfunction are involved in the development of atherosclerosis [5–9]. Increasing evidences have indicated that abnormal migration, extracellular matrix synthesis and proliferation of VSMC can cause atherosclerotic plaque formation [8, 10–12]. However, the underlying mechanism of abnormal migration and proliferation of VSMC remains undetermined.

Long noncoding RNAs (lncRNAs) are one cluster of transcripts greater than 2 hundred nucleotides with limited or no protein-coding capacity. However, they can modulate gene expression at the epigenetic, translation and transcription level [13–17]. Growing studies demonstrated that lncRNAs played roles in several cellular functions such as cell apoptosis, differentiation and proliferation [18–22]. LncRNAs were also deregulated in multiple diseases including tumors, coronary heart disease, scoliosis and intervertebral disc degeneration [23–26]. Recently, a new lncRNA HIX003209 has been identified and found to be invovled in the development of rheumatoid arthritis [27]. Yan et al [27]. showed that HIX003209 expression was increased in peripheral blood mononuclear cells of rheumatoid arthritis patients. Lipopolysaccharide and peptidoglycan increased HIX003209 expression and HIX003209 exaggerated inflammation through regulating miR-6089 expression via Nuclear factor kappa B (NF-kB) / toll like receptor 4 (TLR4) pathway in macrophages. Until now, the function and expression of HIX003209 remains unclear in the development of atherosclerosis.

In our study, we tried to investigate the role of HIX003209 in VSMCs in the development of atherosclerosis. Our results illustrated that HIX003209 played as a critical regulator in atherosclerosis.

## RESULTS

### HIX003209 expression was overexpressed in coronary artery disease

**Figure 1:**
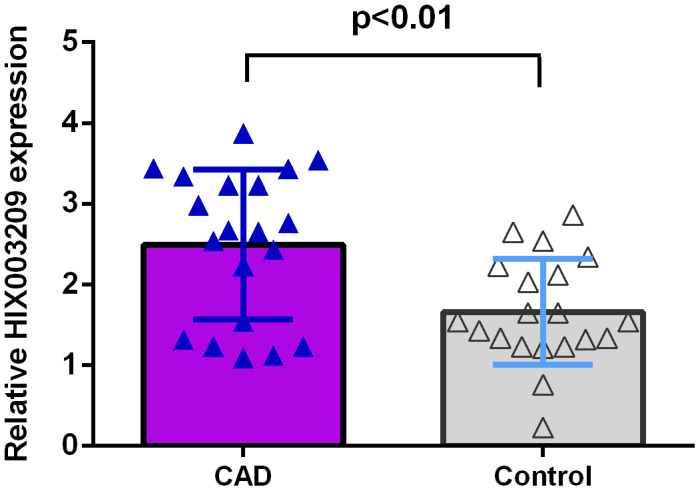
**HIX003209 expression was overexpressed in CAD.** The expression of HIX003209 was higher in atherosclerotic coronary tissues compared to normal coronary artery samples (p<0.01). The data were shown as HIX003209/GADPH.

We firstly detected the expression of HIX003209 in atherosclerotic coronary tissues and normal coronary artery samples. As indicated in Figure 1, the expression of HIX003209 was higher in atherosclerotic coronary tissues compared to normal coronary artery samples (p<0.01).

### HIX003209 was upregulated in VSMCs induced by inflammatory mediators

**Figure 2:**
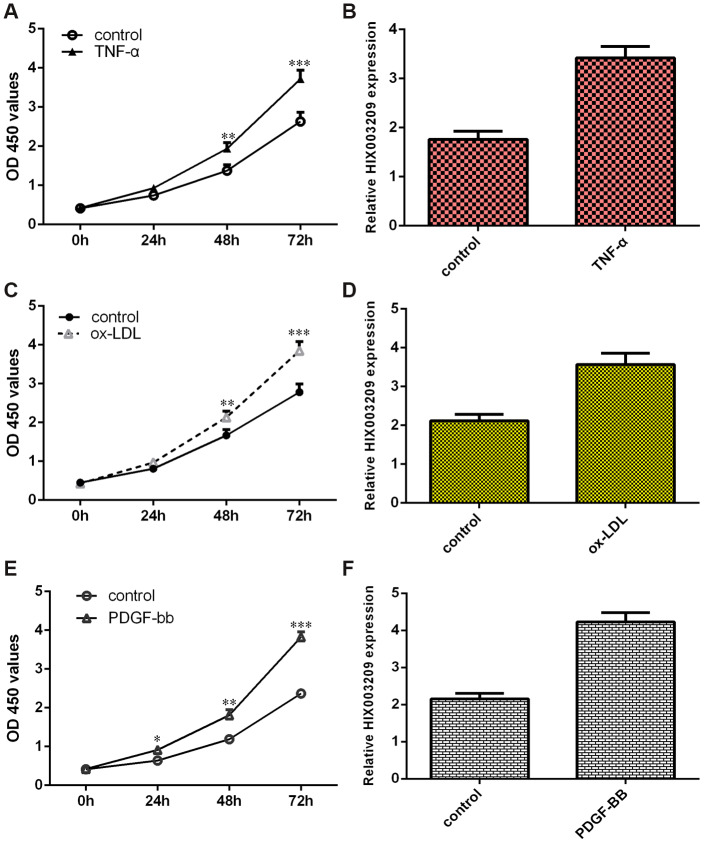
**HIX003209 was upregulated in VSMCs induced by inflammatory mediators.** (**A**) TNF-α promoted VSMC proliferation using CCK-8 method when compare to control group. (**B**) HIX003209 expression was upregulated in VSMCs induced by TNF-α when compare to control group. (**C**) ox-LDL induced VSMC growth using CCK-8 kit when compare to control group. (**D**) The expression of HIX003209 was determined with qRT-PCR analysis. (**E**) PDGF-BB enhanced VSMCs proliferation using CCK-8 kit when compare to control group. (**F**) The expression of HIX003209 was determined with qRT-PCR analysis. *p<0.05, **p<0.01 and ***p<0.001. The data were shown as HIX003209/GADPH.

TNF-α significantly promoted VSMC proliferations compared to the control group (Figure 2A). Then, we showed that HIX003209 expression was upregulated in VSMCs induced by TNF-α (Figure 2B). We also confirmed that ox-LDL induced VSMC growth (Figure 2C). The expression of HIX003209 was higher in VSMCs induced by ox-LDL compared to the control group (Figure 2D). As indicated in Figure 2E, PDGF-BB enhanced VSMCs proliferation when compared to the control group (Figure 2E). The expression of HIX003209 was overexpressed in VSMCs induced by PDGF-BB compared to the control group (Figure 2F).

### Ectopic expression of HIX003209 induced the secretion of inflammatory mediators, cell growth and migration in VSMCs

**Figure 3:**
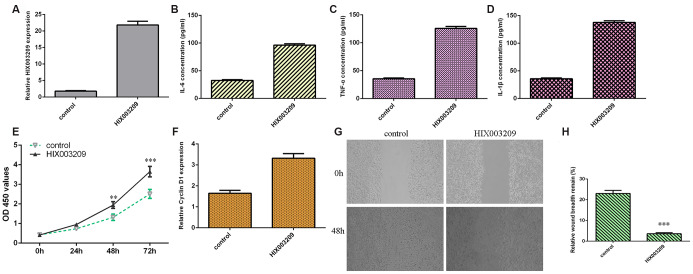
**Ectopic expression of HIX003209 induced inflammatory mediators secretion, cell growth and migration in VSMCs.** (**A**) The expression of HIX003209 was measured with qRT-PCR assay. (**B**) Ectopic expression of HIX003209 induced inflammatory mediators secretion including IL-6 compared to control group. (**C**) The expression of TNF-α in cell culture supernatant was measured by ELISA assay. (**D**) The expression of IL-1β in cell culture supernatant was measured by ELISA assay. (**E**) Elevated expression of HIX003209 promoted VSMCs proliferation compared to control group. (**F**) The expression of cyclin D1 was measured by qRT-PCR analysis. (**G**) Overexpression of HIX003209 induced VSMCs migration compared to control group. (**H**) The relative wound breadth remain of two groups was showed **p<0.01 and ***p<0.001. GAPDH was used as internal control.

HIX003209 was significantly overexpressed in VSMCs after transfection with pcDNA-HIX003209 (Figure 3A). Ectopic expression of HIX003209 induced the secretion of inflammatory mediators including IL-6 (Figure 3B), TNF-α (Figure 3C) and IL-1β (Figure 3D). Elevated expression of HIX003209 promoted VSMCs proliferation (Figure 3E). The expression of cyclin D1 was upregulated in VSMCs after transfection with pcDNA-HIX003209 (Figure 3F). Overexpression of HIX003209 induced VSMCs migration (Figure 3G) and the relative wound breadth remain of two groups was showed in Figure 3H.

### miR-6089 expression was downregulated in CAD

**Figure 4:**
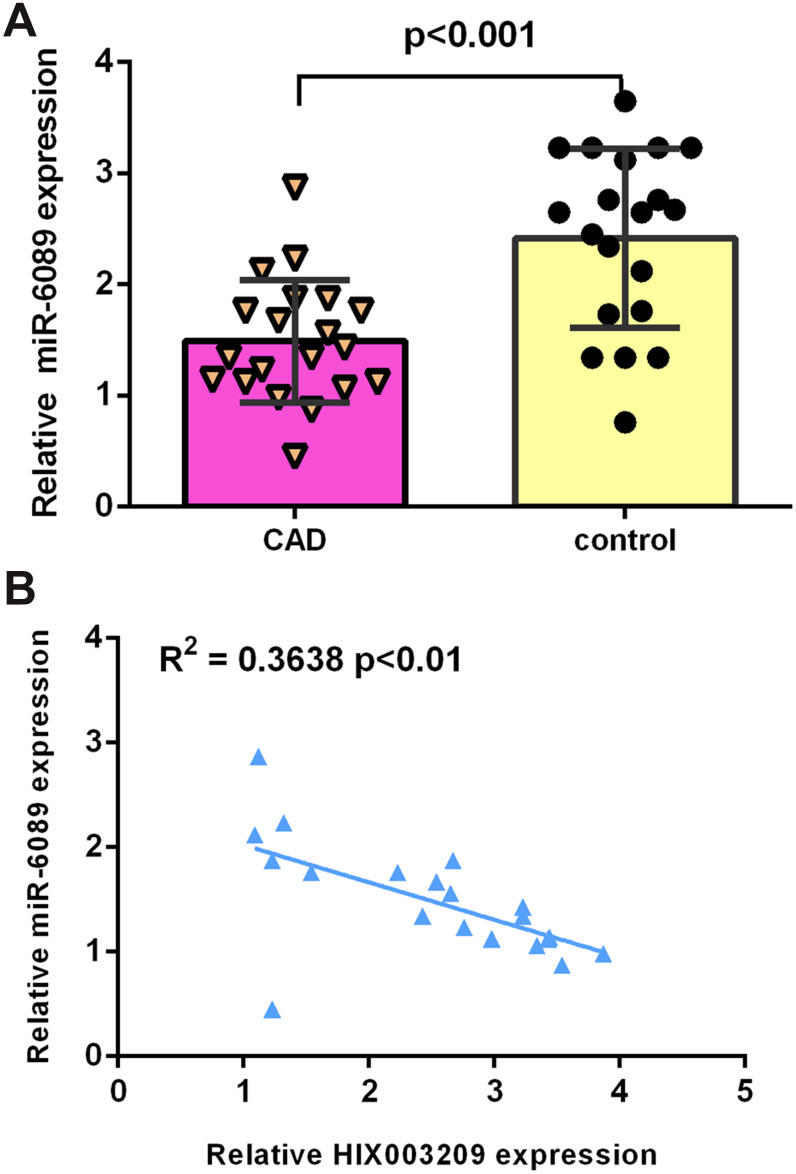
**miR-6089 expression was downregulated in CAD.** (**A**) The expression of miR-6089 was decreased in atherosclerotic coronary tissues compared to normal coronary artery samples. (**B**) There is a negative association between expression of HIX003209 and miR-6089 in atherosclerotic coronary tissues. U6 was used as internal control.

Recent evidence have showed that HIX003209 exaggerates inflammation through sponging miR-6089 in macrophages [27]. We then detected the expression of miR-6089 in atherosclerotic coronary tissues and normal coronary artery samples. As indicated in Figure 4A, the expression of miR-6089 was decreased in atherosclerotic coronary tissues compared to normal coronary artery samples. There was a negative association between the expression of HIX003209 and miR-6089 in atherosclerotic coronary tissues (Figure 4B). Therefore, it is considered that HIX003209 may sponge miR-6089 in VSMCs.

### miR-6089 was downregulated in VSMCs induced by inflammatory mediators

**Figure 5:**
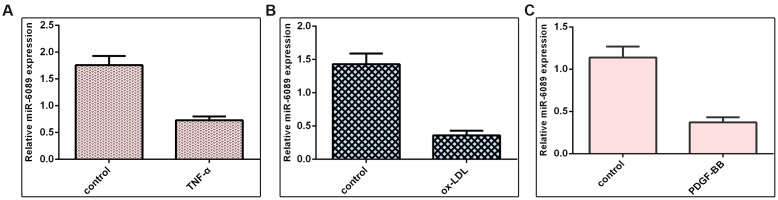
**miR-6089 was downregulated in VSMCs induced by inflammatory mediators.** (**A**) miR-6089 expression was downregulated in VSMCs induced by TNF-α compared to control group. (**B**) ox-LDL can inhibit miR-6089 expression in VSMCs using qRT-PCR analysis compared to control group. (**C**) The expression of miR-6089 expression in VSMCs was determined by qRT-PCR assay. U6 was used as internal control.

We demonstrated that miR-6089 expression was downregulated in VSMCs induced by TNF-α when compared to the control group (Figure 5A). Ox-LDL can inhibit miR-6089 expression in VSMCs when compared to the control group (Figure 5B). Furthermore, we proved that PDGF-BB decreased the expression of miR-6089 expression in VSMCs when compared to the control group (Figure 5C).

### miR-6089 targeted the 3’-UTR of HIX003209

**Figure 6:**
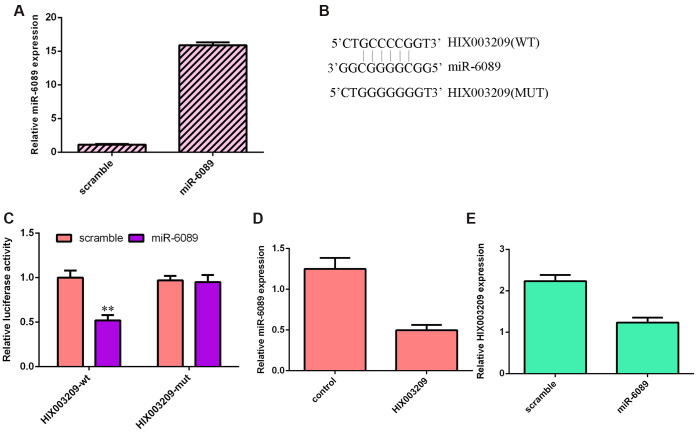
**miR-6089 targets the HIX003209 3’-UTR.** (**A**) The expression of miR-6089 was measured by qRT-PCR analysis. (**B**) There were binding sites between miR-6089 and HIX003209 using Starbase (https://omictools.com/starbase-tool). (**C**) Dual luciferase analysis showed that miR-6089 overexpression decreased luciferase activity of HIX003209 WT-type 3’-UTR but not the mut-type 3’-UTR. (**D**) Overexpression of HIX003209 suppressed the expression of miR-6089 in VSMCs compared to control group. (**E**) Ectopic expression of miR-6089 decreased the HIX003209 expression in VSMCs compared to scramble group.. **p<0.01.

We confirmed that miR-6089 was significantly overexpressed in VSMCs after transfection with miR-6089 mimic (Figure 6A). There were binding sites between miR-6089 and HIX003209 using Starbase (https://omictools.com/starbase-tool) (Figure 6B). Dual luciferase analysis showed that miR-6089 overexpression decreased luciferase activity of HIX003209 WT-type 3’-UTR but not the mut-type 3’-UTR (Figure 6C). Overexpression of HIX003209 suppressed the expression of miR-6089 in VSMCs (Figure 6D). Ectopic expression of miR-6089 decreased HIX003209 expression in VSMCs (Figure 6E).

### HIX003209 induced the secretion of inflammatory mediators, cell growth and migration via regulating miR-6089 expression

**Figure 7:**
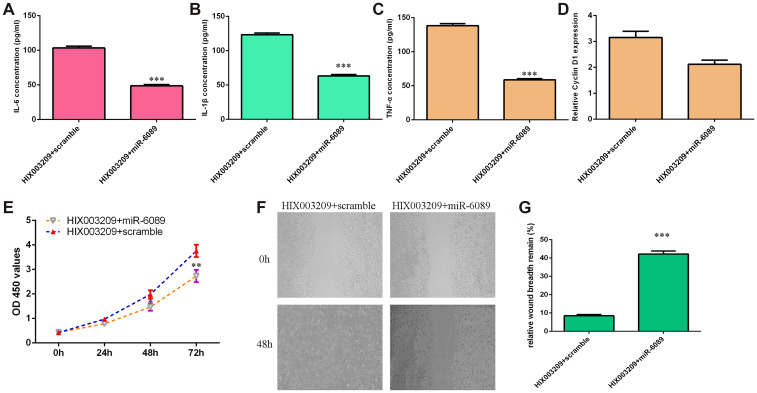
**HIX003209 induced inflammatory mediators secretion, cell growth and migration via regulating miR-6089 expression.** (**A**) The expression of IL-6 was detected by ELISA assay. (**B**) The expression of TNF-α was detected by ELISA assay. (**C**) The expression of IL-1β was detected by ELISA assay. (**D**) The expression of cyclin D1 was measured by qRT-PCR analysis. (**E**) Cell proliferation was measured by CCK-8 analysis. (**F**) miR-6089 overexpression inhibited HIX003209-overexpressing VSMCs migration compared to scramble group. (**G**) The relative wound breadth remain of two groups was showed.**p<0.01 and ***p<0.001.

Rescue test was carried out to study whether HIX003209 induced the secretion of inflammatory mediators, cell growth and migration via regulating miR-6089 expression. qRT-PCR assay data showed that overexpression of miR-6089 decreased inflammatory mediators secretion including IL-6 (Figure 7A), TNF-α (Figure 7B) and IL-1β (Figure 7C) in HIX003209-overexpressing VSMCs when compared to the scramble group. In addition, ectopic expression of miR-6089 inhibited cyclin D1 expression in HIX003209-overexpressing VSMCs when compared to the scramble group (Figure 7D). Elevated expression of miR-6089 suppressed cell proliferation in HIX003209-overexpressing VSMCs when compared to the scramble group (Figure 7E). Furthermore, we indicated that miR-6089 overexpression inhibited HIX003209-overexpressing VSMCs migration (Figure 7F) and the relative wound breadth remain was showed in Figure 7G.

## DISCUSSION

Growing evidences have illustrated that hyper-migration and proliferation of VSMCs was associated with the development of atherosclerosis [28–30]. Therefore, new targets that suppress migration and proliferation of VSMCs may emerge as one effective method for atherosclerosis. This research firstly demonstrated that HIX003209 expression was higher in atherosclerotic coronary tissues compared to the normal coronary artery samples. HIX003209 was upregulated in VSMCs induced by inflammatory mediators including TNF-α, ox-LDL and PDGF-BB. Ectopic expression of HIX003209 promoted cell growth and migration and induced the secretion of inflammatory mediators such as IL-6, TNF-α and IL-1β in VSMCs. Collectively, these results suggested potential role of HIX003209 in modulating VSMCs proliferation, migration and the secretion of inflammatory mediators.

A novel lncRNA HIX003209 has been recently identified to be involved in the development of rheumatoid arthritis [27]. Yan et al [27]. showed that HIX003209 exaggerated inflammation through regulating miR-6089 via NF-kB/TLR4 pathway in macrophages. Increasing evidence have proved that lncRNAs participate in the pathogenesis of cardiovascular diseases. For instance, Wang et al [31]. indicated that ox-LDL and PDGF-BB enhanced LIPCAR expression in VSMCs and LIPCAR overexpression increased VSMCs migration and proliferation and promoted p21, CDK2, PCNA, MMP9, MMP2, Ang-2 and VEGF-A expression. MEG3 modulated apoptosis and proliferation in the ox-LDL-induced VSMCs and acted as one ceRNA for miR-361-5p to regulate ABCA1 expression [32]. However, the function and expression of HIX003209 remains unclear. This research demonstrated that HIX003209 induced VSMCs proliferation, migration and the secretion of inflammatory mediators.

Growing studies have suggested that lncRNA exert its cell biological functions through negative modulation of target miRNAs [33–35]. For example, Liu et al [36]. indicated that GAS5 inhibiting PDGF-bb-treated VSMCs migration and proliferation via regulating miR-21. Tan et al [37]. showed that ANRIL suppressed VSMCs cell senescence through modulating miR-181a expression. LncRNA C2dat1 promoted VSMCs migration and growth via regulating miR-34a-5p expression [38]. Recent research has demonstrated that HIX003209 exaggerates inflammation through sponging miR-6089 in macrophages [27]. We used Starbase to predict that there were binding sites between miR-6089 and HIX003209. MiR-6089 overexpression decreased luciferase activity of HIX003209 WT-type 3’-UTR but not the mut-type 3’-UTR. Furthermore, we showed that miR-6089 was decreased in atherosclerotic coronary tissues compared to the normal coronary artery samples. There is a negative association between expression of HIX003209 and miR-6089 in atherosclerotic coronary tissues. However, the correlation index R2 was only 0.3638 since HIX003209 was only one of the many factors that regulated miR-6089 expression. MiR-6089 expression was downregulated in VSMCs induced by inflammatory mediators including TNF-α, ox-LDL and PDGF-BB. Ectopic expression of HIX003209 induced the secretion of inflammatory mediators, cell growth and migration via regulating miR-6089 expression.

In short, we indicated that HIX003209 expression was higher in atherosclerotic coronary tissues compared to the normal coronary artery samples. Ectopic expression of HIX003209 induced the secretion of inflammatory mediators, cell growth and migration via regulating miR-6089 expression.

## MATERIALS AND METHODS

### Tissues and cell culture and transfection

Atherosclerotic coronary specimens were collected from patients undergoing aortic aneurysm repair surgery and normal coronary arteries were obtained from organ donors from the Second Affiliated Hospital of Dalian Medical University. This research protocol was approved by Ethics Committee of Second Affiliated Hospital of Dalian Medical University. Patients were offered written consent. VSMCs were obtained from ATCC company and were cultured in Dulbecco Modified Eagle Medium (DMEM). PcDNA-HIX003209 and control vectors; miR-6089 mimic or scramble were composed from GenePharma (Shanghai, China) and cell transfection was conducted using Lipofectamine 2000 (Invitrogen, USA). Cells were treated with TNF-α (10 ng/ml) or ox-LDL (50mg/L) or PDGF-BB (20 ng/ml) for 24 hours and HIX003209 and miR-6089 were measured.

### Wound healing and cell proliferation assay

For cell migration assay, wound healing analysis was conducted. Cells were plated in DMEM medium and cell wound was scratched with sterile micropipette tip. Wound migration rate was determined with migration/original distance. Image of the cell wound was captured. For cell proliferation analysis, cell counting kit-8 (CCK-8) analysis kit (Dojindo, Kumamoto, Japan) was used. Cells were cultured in 96 well dishes and continued to plate for 72 hours. The absorbance optical density at 450 nm was recorded.

### ELISA (Enzyme-linked immunosorbent assay)

Protein level of IL-6, TNF-α and IL-1β in the cell culture supernatant was detected by using ELISA based on standard process of ELISA (R&D Systems, USA).

### Quantitative real-time PCR

Total RNA was separated from clinical specimens and treated cells using TRIzol kit (Invitrogen). The miRNA, lncRNA and mRNA expression was quantified by using Quantitative PCR assay with SYBR Green mix on the Bio-Rad IQ5 PCR system (Hercules, USA) following to standard protocol. Relative gene expression was calculated by 2^-ΔΔCq^ method. GAPDH and U6 gene expression was performed as the internal control for HIX003209, Cyclin D1 and miR6089 respectively. The primer sequences were listed as following: HIX003209, 5’-ACTGCTCGCCAGAACACTAC-3’, and 5’-GGTGAGGTTGATCGGGGTTT-3’; GAPDH, 5’-CTGACTTCAACAGCGACACC-3’ and 5’-GTGGTCCAGGGGTCTTACTC-3’; miR6089, 5'-GGAGGCCGGGGTGGGGCG-3'; Cyclin D1, 5'-AACTACCTGGACCGCTTCCT-3' and 5'-CCACTTGAGCTTGTTCACCA-3'.

### Luciferase gene reporter analysis

The miR-6089 (mutant type and wild type) sequences of HIX003209 3’ untranslated region (3’-UTR) were amplified with PCR and cloned into pGL3 reporter vector (Promega, USA). For luciferase reporter analysis, cells were cultured on the 24 well dishes. Cells were cotransfected by miR-6089 and scramble and pGL3 reporter constructs by Lipofectamine 2000 kit following to manufacturer's information. The relative luciferase ability normalized to the Renilla luciferase value.

### Statistical analysis

Results were indicated as mean ±standard Deviation and statistical analysis was evaluated by SPSS (version 16.0, SPSS, Chicago, IL, USA). Student’s t test was utilized to evaluate group significant difference. Statistically significant was set as P < 0.05.

## References

[1] Wei Y, Nazari-Jahantigh M, Neth P, Weber C, Schober A. MicroRNA-126, -145, and -155: a therapeutic triad in atherosclerosis? Arterioscler Thromb Vasc Biol. 2013; 33:449–54. 10.1161/ATVBAHA.112.300279 23324496

[2] Xue Y, Wei Z, Ding H, Wang Q, Zhou Z, Zheng S, Zhang Y, Hou D, Liu Y, Zen K, Zhang CY, Li J, Wang D, Jiang X. MicroRNA-19b/221/222 induces endothelial cell dysfunction via suppression of PGC-1α in the progression of atherosclerosis. Atherosclerosis. 2015; 241:671–81. 10.1016/j.atherosclerosis.2015.06.031 26117405

[3] Zhang C. MicroRNA and vascular smooth muscle cell phenotype: new therapy for atherosclerosis? Genome Med. 2009; 1:85. 10.1186/gm85 19744308 PMC2768992

[4] Rayner KJ, Sheedy FJ, Esau CC, Hussain FN, Temel RE, Parathath S, van Gils JM, Rayner AJ, Chang AN, Suarez Y, Fernandez-Hernando C, Fisher EA, Moore KJ. Antagonism of miR-33 in mice promotes reverse cholesterol transport and regression of atherosclerosis. J Clin Invest. 2011; 121:2921–31. 10.1172/JCI57275 21646721 PMC3223840

[5] Spiroglou SG, Kostopoulos CG, Varakis JN, Papadaki HH. Adipokines in periaortic and epicardial adipose tissue: differential expression and relation to atherosclerosis. J Atheroscler Thromb. 2010; 17:115–30. 10.5551/jat.1735 20145358

[6] Shyu KG, Chen SC, Wang BW, Cheng WP, Hung HF. Mechanism of the inhibitory effect of atorvastatin on leptin expression induced by angiotensin II in cultured human coronary artery smooth muscle cells. Clin Sci (Lond). 2012; 122:33–42. 10.1042/CS20110114 21806545

[7] Shin HJ, Oh J, Kang SM, Lee JH, Shin MJ, Hwang KC, Jang Y, Chung JH. Leptin induces hypertrophy via p38 mitogen-activated protein kinase in rat vascular smooth muscle cells. Biochem Biophys Res Commun. 2005; 329:18–24. 10.1016/j.bbrc.2004.12.195 15721267

[8] Li P, Zhu N, Yi B, Wang N, Chen M, You X, Zhao X, Solomides CC, Qin Y, Sun J. MicroRNA-663 regulates human vascular smooth muscle cell phenotypic switch and vascular neointimal formation. Circ Res. 2013; 113:1117–27. 10.1161/CIRCRESAHA.113.301306 24014830 PMC4537615

[9] Rajesh M, Mukhopadhyay P, Haskó G, Huffman JW, Mackie K, Pacher P. CB2 cannabinoid receptor agonists attenuate TNF-alpha-induced human vascular smooth muscle cell proliferation and migration. Br J Pharmacol. 2008; 153:347–57. 10.1038/sj.bjp.0707569 17994109 PMC2219520

[10] Quintavalle M, Elia L, Condorelli G, Courtneidge SA. MicroRNA control of podosome formation in vascular smooth muscle cells in vivo and in vitro. J Cell Biol. 2010; 189:13–22. 10.1083/jcb.200912096 20351064 PMC2854384

[11] Marquart TJ, Wu J, Lusis AJ, Baldán Á. Anti-miR-33 therapy does not alter the progression of atherosclerosis in low-density lipoprotein receptor-deficient mice. Arterioscler Thromb Vasc Biol. 2013; 33:455–58. 10.1161/ATVBAHA.112.300639 23288159 PMC3587119

[12] Li P, Liu Y, Yi B, Wang G, You X, Zhao X, Summer R, Qin Y, Sun J. MicroRNA-638 is highly expressed in human vascular smooth muscle cells and inhibits PDGF-BB-induced cell proliferation and migration through targeting orphan nuclear receptor NOR1. Cardiovasc Res. 2013; 99:185–93. 10.1093/cvr/cvt082 23554459 PMC3687750

[13] Zou Y, Zhong Y, Wu J, Xiao H, Zhang X, Liao X, Li J, Mao X, Liu Y, Zhang F. Long non-coding PANDAR as a novel biomarker in human cancer: A systematic review. Cell Prolif. 2018; 51:e12422. 10.1111/cpr.12422 29226461 PMC6528858

[14] Zhuang Y, Jiang H, Li H, Dai J, Liu Y, Li Y, Miao L, Cai H, Xiao Y, Xia H, Wang Y, Shi M. Down-regulation of long non-coding RNA AFAP1-AS1 inhibits tumor cell growth and invasion in lung adenocarcinoma. Am J Transl Res. 2017; 9:2997–3005. 28670387 PMC5489899

[15] Zhu S, Fu W, Zhang L, Fu K, Hu J, Jia W, Liu G. LINC00473 antagonizes the tumour suppressor miR-195 to mediate the pathogenesis of Wilms tumour via IKKα. Cell Prolif. 2018; 51:e12416. 10.1111/cpr.12416 29159834 PMC6528909

[16] Zhou DD, Liu XF, Lu CW, Pant OP, Liu XD. Long non-coding RNA PVT1: emerging biomarker in digestive system cancer. Cell Prolif. 2017; 50:e12398. 10.1111/cpr.12398 29027279 PMC6529066

[17] Yu X, Zheng H, Tse G, Chan MT, Wu WK. Long non-coding RNAs in melanoma. Cell Prolif. 2018; 51:e12457. 10.1111/cpr.12457 29582492 PMC6528844

[18] Zheng J, Yi D, Liu Y, Wang M, Zhu Y, Shi H. Long nonding RNA UCA1 regulates neural stem cell differentiation by controlling miR-1/Hes1 expression. Am J Transl Res. 2017; 9:3696–704. 28861160 PMC5575183

[19] Zhao J, Zhang C, Gao Z, Wu H, Gu R, Jiang R. Long non-coding RNA ASBEL promotes osteosarcoma cell proliferation, migration, and invasion by regulating microRNA-21. J Cell Biochem. 2018; 119:6461–69. 10.1002/jcb.26671 29323740

[20] Zhang Y, Dang YW, Wang X, Yang X, Zhang R, Lv ZL, Chen G. Comprehensive analysis of long non-coding RNA PVT1 gene interaction regulatory network in hepatocellular carcinoma using gene microarray and bioinformatics. Am J Transl Res. 2017; 9:3904–17. 28979669 PMC5622238

[21] Zhang J, Yin M, Peng G, Zhao Y. CRNDE: an important oncogenic long non-coding RNA in human cancers. Cell Prolif. 2018; 51:e12440. 10.1111/cpr.12440 29405523 PMC6528921

[22] Yu X, Zheng H, Tse G, Zhang L, Wu WK. CASC2: an emerging tumour-suppressing long noncoding RNA in human cancers and melanoma. Cell Prolif. 2018; 51:e12506. 10.1111/cpr.12506 30094876 PMC6528875

[23] Yang C, Wu K, Wang S, Wei G. Long non-coding RNA XIST promotes osteosarcoma progression by targeting YAP via miR-195-5p. J Cell Biochem. 2018; 119:5646–56. 10.1002/jcb.26743 29384226

[24] Xu R, Feng F, Yu X, Liu Z, Lao L. LncRNA SNHG4 promotes tumour growth by sponging miR-224-3p and predicts poor survival and recurrence in human osteosarcoma. Cell Prolif. 2018; 51:e12515. 10.1111/cpr.12515 30152090 PMC6528889

[25] Li Z, Li X, Chen C, Li S, Shen J, Tse G, Chan MT, Wu WK. Long non-coding RNAs in nucleus pulposus cell function and intervertebral disc degeneration. Cell Prolif. 2018; 51:e12483. 10.1111/cpr.12483 30039593 PMC6528936

[26] Chen C, Tan H, Bi J, Li L, Rong T, Lin Y, Sun P, Liang J, Jiao Y, Li Z, Sun L, Shen J. LncRNA-SULT1C2A regulates Foxo4 in congenital scoliosis by targeting rno-miR-466c-5p through PI3K-ATK signalling. J Cell Mol Med. 2019; 23:4582–91. 10.1111/jcmm.14355 31044535 PMC6584475

[27] Yan S, Wang P, Wang J, Yang J, Lu H, Jin C, Cheng M, Xu D. Long Non-coding RNA HIX003209 Promotes Inflammation by Sponging miR-6089 via TLR4/NF-κB Signaling Pathway in Rheumatoid Arthritis. Front Immunol. 2019; 10:2218. 10.3389/fimmu.2019.02218 31620132 PMC6759987

[28] Zhang Y, Wang Y, Wang X, Zhang Y, Eisner GM, Asico LD, Jose PA, Zeng C. Insulin promotes vascular smooth muscle cell proliferation via microRNA-208-mediated downregulation of p21. J Hypertens. 2011; 29:1560–68. 10.1097/HJH.0b013e328348ef8e 21720271

[29] Sun Y, Chen D, Cao L, Zhang R, Zhou J, Chen H, Li Y, Li M, Cao J, Wang Z. MiR-490-3p modulates the proliferation of vascular smooth muscle cells induced by ox-LDL through targeting PAPP-A. Cardiovasc Res. 2013; 100:272–79. 10.1093/cvr/cvt172 23821525

[30] Stein JJ, Iwuchukwu C, Maier KG, Gahtan V. Thrombospondin-1-induced vascular smooth muscle cell migration and proliferation are functionally dependent on microRNA-21. Surgery. 2014; 155:228–33. 10.1016/j.surg.2013.08.003 24314882

[31] Wang X, Li D, Chen H, Wei X, Xu X. Expression of Long Noncoding RNA LIPCAR Promotes Cell Proliferation, Cell Migration, and Change in Phenotype of Vascular Smooth Muscle Cells. Med Sci Monit. 2019; 25:7645–51. 10.12659/MSM.915681 31603865 PMC6800467

[32] Wang M, Li C, Zhang Y, Zhou X, Liu Y, Lu C. LncRNA MEG3-derived miR-361-5p regulate vascular smooth muscle cells proliferation and apoptosis by targeting ABCA1. Am J Transl Res. 2019; 11:3600–09. 31312370 PMC6614649

[33] Pan Y, Wu Y, Hu J, Shan Y, Ma J, Ma H, Qi X, Jia L. Long noncoding RNA HOTAIR promotes renal cell carcinoma malignancy through alpha-2, 8-sialyltransferase 4 by sponging microRNA-124. Cell Prolif. 2018; 51:e12507. 10.1111/cpr.12507 30105850 PMC6528863

[34] Ma X, Qi S, Duan Z, Liao H, Yang B, Wang W, Tan J, Li Q, Xia X. Long non-coding RNA LOC554202 modulates chordoma cell proliferation and invasion by recruiting EZH2 and regulating miR-31 expression. Cell Prolif. 2017; 50:e12388. 10.1111/cpr.12388 28963737 PMC6529120

[35] Liu J, Song Z, Feng C, Lu Y, Zhou Y, Lin Y, Dong C. The long non-coding RNA SUMO1P3 facilitates breast cancer progression by negatively regulating miR-320a. Am J Transl Res. 2017; 9:5594–602. 29312511 PMC5752909

[36] Liu K, Liu C, Zhang Z. lncRNA GAS5 acts as a ceRNA for miR-21 in suppressing PDGF-bb-induced proliferation and migration in vascular smooth muscle cells. J Cell Biochem. 2019; 120:15233–40. 10.1002/jcb.28789 31069831

[37] Tan P, Guo YH, Zhan JK, Long LM, Xu ML, Ye L, Ma XY, Cui XJ, Wang HQ. LncRNA-ANRIL inhibits cell senescence of vascular smooth muscle cells by regulating miR-181a/Sirt1. Biochem Cell Biol. 2019; 97:571–80. 10.1139/bcb-2018-0126 30789795

[38] Wang H, Jin Z, Pei T, Song W, Gong Y, Chen D, Zhang L, Zhang M, Zhang G. Long noncoding RNAs C2dat1 enhances vascular smooth muscle cell proliferation and migration by targeting MiR-34a-5p. J Cell Biochem. 2019; 120:3001–08. 10.1002/jcb.27070 30474870

